# Urine polymerase chain reaction tests: stewardship helper or hinderance?

**DOI:** 10.1017/ash.2024.71

**Published:** 2024-03-06

**Authors:** Jessica Zering, Erica J. Stohs

**Affiliations:** 1 Healthcare Associated Infections Program, Washington State Department of Health, Shoreline, WA, USA; 2 Division of Infectious Diseases, Department of Medicine, University of Nebraska Medical Center, Omaha, NE, USA

## Abstract

Urine polymerase chain reaction is a laboratory test promoted to healthcare professionals working in long-term care facilities as a rapid diagnostic platform for urinary tract infection. Little is known about the place of this testing and its potential impact on antimicrobial stewardship programs. In this commentary, we review the currently available literature and provide recommendations for long-term care stewardship programs to consider.

Urine polymerase chain reaction (PCR) is a laboratory test promoted to healthcare professionals working in long-term care facilities (LTCFs) as a rapid diagnostic platform for urinary tract infection (UTI). Interactions with personnel implementing antimicrobial stewardship (AS) in LTCF prompted inquiries regarding the place of urine PCR testing within the context of AS. Rapid diagnostics can facilitate stewardship through timely identification of an infective organism, but this typically occurs when antimicrobial stewards optimize their utilization.^1,2^ The introduction of urine PCR in non-acute care settings has occurred independent of AS as many facilities’ AS programs are nascent. Here, we explore the considerations of urine PCR in the context of diagnostic stewardship.

Antibiotic prescribing for UTI is an important AS target as it represents the most common indication for antibiotics in LTCFs.^3,4^ Up to 50% of antibiotics prescribed for UTI in this setting are inappropriate.^3,4^ Asymptomatic bacteriuria (ASB) is commonly misdiagnosed as a UTI in older adults, especially postmenopausal women,^3^ leading to overuse of antibiotics, increased risk of antibiotic resistance, and adverse events such as *C. difficile* infection.^3,4^ The prevalence of ASB is estimated to be as high as 50% for residents of LTCFs.^3,5^

## What is urine polymerase chain reaction?

Urine PCR is a laboratory-developed, non-FDA-approved multiplex testing method that uses pathogen-specific primers to detect bacterial organisms and some antibiotic resistance genes.^6,7^ Laboratory-developed tests are not required to undergo formal FDA approval processes, as they are overseen by individual Clinical Laboratory Improvement Amendments (CLIA)-certified laboratories.^8^ PCR testing is unique in that it can detect the genetic information of non-viable organisms.^9^ Manufacturers report bacterial detection either semi-quantitatively or qualitatively,^7,10–12^ the former displayed as cells/mL or copies/µL.^10–12^ Next-generation sequencing is available as a subset of urine PCR testing, which is beyond the scope of this review^7^. Urine PCR is marketed as having a rapid turn-around time and increased sensitivity compared to standard urine culture (SUC) techniques.^7^ Because urine PCR cannot provide phenotypic antibiotic susceptibilities, it requires additional cost in addition to that of SUC.^7^ At present, we found five manufacturers offering urine PCR based on literature review and web search (see Supplemental Table 1).

## Threats of urine polymerase chain reaction on antimicrobial stewardship

Although PCR testing methods have been successfully utilized for other infectious diseases (eg, COVID-19, *C. difficile*), there are limitations related to its use and design for urine testing that impact AS programs. First, studies evaluating urine PCR do not assure reliable clinical diagnostic criteria for UTI are met prior to urine sampling or assure quality of the urine specimen obtained. Second, urine PCR results can be confusing because they are overly sensitive and difficult to interpret. Finally, there is insufficient unbiased evidence to demonstrate that urine PCR improves clinical outcomes.

### Right patient, right specimen

Best practice criteria exist to guide diagnosis and treatment of UTI, including IDSA and Loeb Minimum Criteria for UTI (see Supplemental Table 2).^3,5,13,14^ Despite the existence of these criteria, dogmas surrounding UTI often lead to inappropriate urine sampling. Cloudy or malodorous urine, falls, and confusion are commonly mistaken for symptoms of a UTI.^5^

Studies evaluating urine PCR did not utilize best practice criteria for UTI. A single-site, non-inferiority study performed in the outpatient setting of SUC techniques versus PCR included participants ≥ 60 years of age reporting non-specific symptoms such as urinary incontinence, cloudy or strong-smelling urine, and agitation.^15^ Another study examined all urinary specimens with bacteriuria of ≥ 10^4^ CFUs/mL without accounting for presence of clinical presentation.^16^ Van der zee et al collected urine specimens from 211 patients in the hospital and outpatient settings with and without catheters in whom a UTI was suspected but not confirmed using validated definitions.^17^

Obtaining a quality urine specimen is also important, as contamination can occur when proper technique is not followed.^5^ Although molecular urine diagnostics should theoretically assure appropriate urine sampling, one manufacturer advertises the option of swabbing the briefs of residents who are unable to give a clean-catch sample.^7,18^ This technique is not an acceptable practice, nor is it endorsed by societies’ guidelines.^3,5,13,14^

### Confusing results

*Escherichia coli* causes 75–95% of uncomplicated UTIs, with other common organisms including *Klebsiella pneumoniae*, *Proteus mirabilis*, and *Staphylococcus saprophyticus*.^13^ Multiplex urine PCR panels assess samples for between 9 and 31 different bacteria and some *Candida spp.* (see Supplemental Table 1), arguably more targets than necessary.^9,15,16^ Users may be confused with urine PCR results which are reported as organisms per quantity of urine, that is, cells/mL or copies/µL.^10–12^ The standard colony-forming units (CFUs)/mL reported with SUC considers only those cells that can actively divide under specified conditions. There is a gap in data to provide guidance on the interpretation of quantitative urine PCR results reported as cells/mL or copies/µL per organism.^7,9–12^

Urine PCR testing is more sensitive than SUC techniques, meaning that it is more likely to generate a positive result for organisms that do not represent the infectious agent.^6,7,9,15,17^ It is only minimally superior for the detection of *E. coli*.^7,15,16^ In a non-inferiority trial, 29% of PCR samples grew *E. coli* compared to 34% of the same samples utilizing SUC techniques.^15^ A small prospective study comparing the results of urine samples using both PCR and SUC techniques showed that 64% of PCR samples were positive for *E. coli* compared to 58% of SUC samples.^16^ A single-site, non-inferiority study performed in the outpatient setting collected urine samples for SUC and PCR testing from participants ≥ 60 years of age.^15^ Of 582 patients sampled, 56% had a positive urine PCR result versus 37% with positive culture result; among the 217 with a positive urine culture, there was 90% agreement with urine PCR, suggesting that clinicians are not missing causative UTI organisms with culture alone.^15^ Many of the discordant results were organisms not commonly thought to be causative UTI organisms, such as *Actinobaculum schaalii* and coagulase-negative Staphylococci.^15^

### Lack of unbiased clinical outcomes

Molecular detection of urinary organisms lacks evidence showing improved patient outcomes.^6^ A systematic review and meta-analysis included six studies comparing urine PCR to urine culture.^6^ None assessed patient outcomes, specifically symptom response to antibiotics started due to urine PCR testing results.^6^ Moreover, they concluded that the six studies carried high risk of bias due to manufacturer funding sources.^6^ Since publication, one observational study claimed an association between the use of PCR plus antibiotic susceptibility testing and a reduction in emergency department utilization and hospital admissions; however, the study population was identified retrospectively by using diagnostic codes for infections of the genitourinary tract and dysuria.^19^ Molecular testing in patients with poorly defined urinary symptoms lead to misdiagnoses and overtreatment, which can lead to harms associated with antibiotic overuse.^3,6,20–23^

## Potential benefits of urine polymerase chain reaction

There are notable stewardship limitations with urine PCR and opportunities for further study. PCR demonstrated an increased sensitivity for *Ureaplasma urealyticum, Mycoplasma spp*., and *Aerococcus urinae*, organisms that cause UTI in highly specialized and rare circumstances.^6,15^ Clinical outcomes associated with this testing could be studied in individuals who have a lengthy history of confirmed dysuria and UTIs unresponsive to treatment; however, Mycoplasma- and Ureaplasma-specific PCR platforms exist for patients in whom clinicians have a high index of suspicion for these organisms. Moreover, the setting for such study would not include LTCF.

## Conclusion

Clinicians should utilize caution with adopting urine PCR for diagnosis of UTI. Data associated with this newly advertised diagnostic modality carry considerable limitations and bias.^6,19^ PCR cannot replace SUC techniques.^6,7,9,16^ Healthcare facilities considering urine PCR can work with laboratory and AS personnel to optimize diagnostic stewardship practices for UTI. Antibiograms should be implemented to ensure immediate and proper empiric coverage for true UTIs. More data utilizing strict best practice definitions of UTI and primers focused on organisms of clinical significance per clinical guidelines are needed to determine if urine PCR has a place within a UTI diagnosis. For now, education focused on appropriate testing and sampling for UTI remains a key intervention for both providers and patients in settings that regularly work with the older adult population.

## Supporting information

Zering and Stohs supplementary material Zering and Stohs supplementary material

## Supplementary material

For supplementary material accompanying this paper visit http://doi.org/10.1017/ash.2024.71. click here to view supplementary material
