# Supplementary material for: Urine polymerase chain reaction tests: stewardship helper or hinderance?
Source: Antimicrob Steward Healthc Epidemiol. 2024 Mar 6;4(1):e77. doi: 10.1017/ash.2024.71 (PMC11077600; doi:10.1017/ash.2024.71)
Supplement: Zering and Stohs supplementary material [file S2732494X24000718sup001.docx]

**Table 1: Available Testing Panels***

|  | **Pathnostics Guidance® UTI Panel^11^** | **GENETWORx Molecular PCR UTI Test**^17^ | **Vikor Urine-ID™**^10^ | **Solaris Diagnostics UTI ID Panel**^23^ | **Precision Life Sciences Molecular UTI Test**^9^ |
| --- | --- | --- | --- | --- | --- |
| **Gram-Negative Targets** | *Acinetobacter baumannii,*  *Citrobacter freundii,*  *Citrobacter koseri,*  *Escherichia coli,*  *Klebsiella oxytoca,*  *Klebsiella pneumoniae,*  *Morganella morganii,*  *Pantoea agglomerans,*  *Proteus mirabilis,*  *Providencia stuartii,*  *Pseudomonas aeruginosa,*  *Serratia marcescens* | *Acinetobacter baumannii,*  *Bacteroides fragilis,*  *Enterobacter cloacae,*  *Escherichia coli,*  *Klebsiella oxytoca,*  *Klebsiella pneumoniae,*  *Morganella morganii,*  *Proteus mirabilis,*  *Pseudomonas aeruginosa,*  *Serratia marcescens* | *Acinetobacter baumannii,*  *Citrobacter freundii,*  *Enterobacter cloacae,*  *Haemophilus ducreyi,*  *Klebsiella oxytoca,*  *Klebsiella pneumoniae, Morganella morganii,*  *Neisseria gonorrhoeae,*  *Prevotella bivia,*  *Proteus mirabilis,*  *Proteus vulgaris,*  *Providencia stuartii,*  *Pseudomonas aeruginosa,*  *Serratia marcescens* | *Escherichia coli,*  *Enterobacter cloacae,*  *Proteus mirabilias,*  *Klebsiella pneumoniae,*  *Morganella morganii,*  *Serratia marcescens,*  *Klebsiella oxytoca,*  *Providencia stuartii, Pseudomonas aeruginosa* | *Citrobacter freundii, Citrobacter koseri,*  *Enterobacter aerogenes, Enterobacter cloacae,*  *Escherichia coli,*  *Klebsiella oxytoca,*  *Klebsiella pneumoniae,*  *Morganella morgani,*  *Pantoea agglomerans,*  *Proteus mirabilis,*  *Proteus vulgaris,*  *Providencia stuartii,*  *Pseudomonas aeruginosa,*  *Serratia marcescens* |
| **Gram-Positive Bacterial Targets** | *Actinotignum schaalii,*  *Aerococcus urinae,*  *Alloscardovia omnicolens,*  *Corynebacterium riegelii,*  *Enterococcus faecalis,*  *Enterococcus faecium,*  *Staphylococcus aureus,*  *Streptococcus agalactiae* | *Enterococcus faecalis,*  *Enterococcus faecium,*  *Streptococcus agalactiae,*  *Staphylococcus aureus,*  *Staphylococcus epidermidis* | *Actinobaculum schaalii*  *Aerococcus urinae*  *Alloscardovia omnicolens*  *Atopobium vaginae,*  *Corynebacterium riegelii,*  *Corynebacterium urealyticum,*  *Enterococcus faecalis,*  *Enterococcus faecium,*  *Staphylococcus aureus,*  *Staphylococcus epidermidis,*  *Staphylococcus saprophyticus* | *Staphylococcus aureus,*  *Staphylococcus*  *Saprophyticus,*  *Enterococcus faecalis,*  *Streptococcus agalactiae* | *Actinobaculum schaalii,*  *Aerococcus urinae,*  *Alloscardovia omnicolens,*  Coagulase Negative Staphylococci,  *Enterococcus faecalis, Enterococcus faecium,*  *Staphylococcus aureus,*  *Streptococcus agalactiae,*  Viridans Group Streptococci |
| **Fungal Targets** | *Candida albicans,*  *Candida auris,*  *Candida glabrata,*  *Candida parapsilosis,* | *Candida albicans,*  *Candida glabrata,*  *Candida krusei,*  *Candida parapsilosis,*  *Candida tropicalis* | *Candida albicans,*  *Candida glabrata,*  *Candida krusei,*  *Candida parapsilosis*  *Candida tropicalis* | *Candida* spp. | *Candida albicans Candida auris Candida glabrata Candida parapsilosis* |
| **Gram-Variable or Atypical Organism Targets** | *Gardnerella vaginalis,*  *Mycoplasma hominis,*  *Ureaplasma urealyticum* |  | *Gardnerella vaginalis,*  *Mobiluncus curtisii, Mobiluncus mulieris,*  *Mycoplasma genitalium, Mycoplasma hominis,*  *Treponema pallidum* (Syphilis),  *Trichomonas vaginalis,*  *Ureaplasma urealyticum* | *Ureaplasma urealyticum,*  *Mycoplasma hominis* | *Mycoplasma hominis,*  *Ureaplasma urealyticum* |
| **Antibiotic Resistance Gene Classes/Types Detected** | Ampicillin  Carbapenem  ESBL  Methicillin  Fluoroquinolones  Vancomycin | Aminoglycosides  Carbapenems  ESBL  Macrolide  Tetracycline  Fluoroquinolone  Vancomycin  Methicillin | Aminoglycosides  Beta-Lactams  Vancomycin  Macrolides  Carbapenems  Methicillin  Polymyxins  Fluoroquinolones  Sulfonamides  Tetracycline | Unknown | Vancomycin |

*Based upon information available from the manufacturer’s website

Abbreviations: ESBL, extended-spectrum beta lactamases

**Table 2: Standard Best Practice Definitions for Urinary Tract Infections**^3,5,12,13^

| **Urinary Tract Infection**^5,12^ | **Asymptomatic Bacteriuria (Non-Catheterized Patients)**^5^ | **Loeb Minimum Criteria for Non-Catheterized Patients**^3,13^ | **Loeb Minimum Criteria for Catheterized Patients**^3,13^ |
| --- | --- | --- | --- |
| A positive urinalysis plus symptoms, which can include:   - Urgency - Frequency - Dysuria - Suprapubic tenderness - Gross hematuria - Costovertebral tenderness - Fever ≥ 100.4⁰F | 1 or more species of bacteria growing in the urine at (≥ 100,000 CFU/mL or (≥ 100,000,000 CFU/L, regardless of pyuria and without signs and symptoms of a urinary tract infection | Acute dysuria alone or fever (100⁰F or 2.4⁰F over baseline)  Plus at least one of the following:   - Frequency - Gross hematuria - Suprapubic tenderness - Costovertebral angle tenderness - New or worsening urinary incontinence - Urgency | At least one of the following:   - Rigors - Fever (100⁰F or 2.4⁰F over baseline) - New costovertebral tenderness - New onset of delirium |

Abbreviations: CFU, colony-forming units; mL, milliliter; L, liter; F, Fahrenheit
